# Surgical Clinic at Harlem Hospital, New York

**Published:** 1893-03

**Authors:** Thomas H. Manley


ATLANTA
MEDICAL AND SURGICAL JOURNAL.

VOL. X. MARCH, 1893. NO. 1.

EDITED BY
LUTHER B. GRANDY, M. D., AND MILLER B. HUTCHINS, M. D.,
WITH THE CO-OPERATION OF
H. V. M. MILLER, M. D., LL. D., VIRGIL O. HARDON, M. D.,
FLOYD W. McRAE, M. D., AND ARTHUR G. HOBBS, M. D.

### *CLINICAL LECTURE.*

### SURGICAL CLINIC AT HARLEM HOSPITAL, NEW YORK.

By THOMAS H. MANLEY, M. D.

### (1) URANOPLASTY. (2) OSTEOTOMY OF ULNA FOR DEFORMITY FOLLOWING FRACTURE. (3) PLASTIC OPERATION ON THE HAND FOR CONTRACTURE FOLLOWING A BURN.

Of the first case Dr. Manley said the history was as follows: A female, age 21, single, no organic disease, general health good at the present time. A little more than a year ago she contracted syphilis, but was not regularly treated. Six months ago patient commenced to notice that the soft palate was tender and very sensitive to the passage, through mouth, of any hot or cold liquids. This was soon followed by a marked nasal element in speech, with a tendency, when she swallowed, of the fluids making their way into the nose and coming through the ears. She said, when she entered, that the opening which she had discovered through the roof of her mouth had become lately rapidly larger. Said she entered the hospital to have an operation performed for its cure. The speaker said that before he commenced to operate, he would say that an operation for the cure of a perforation in the soft or hard palate was always one difficult of performance. Much easier in the adult than in the young child, however. If a pulmonary anesthetic were given the difficulties were quite insurmountable. As soon as commenced the ether-cone is removed and we begin the tedious, necessary dissection. The patient regains consciousness and resists us ; the blood leaves the vascular parts, issues on division of the tissues, in great quantities, strangulating the patient and obscuring the operative field. The patient must be repeatedly anesthetized. Indeed, by the older methods of operating a uranoplasty was one of the most trying in surgery. As to the technique of operation, he said that one must commence by thoroughly detaching and slackening all the palatal muscles; then reflush the orifice, which in this case would admit two fingers; and, lastly, bring the parts together and fix them. He had, he said, already once operated on this case, the patient taking no anesthetic; at which time the operation proved full of difficulties and was very bloody and unsatisfactory. The result was *nil.* Now, after an interval of two weeks, he would again attempt it, this time employing cocaine and analgesia.

After amply cocainizing the parts by surface and hypodermic employment of the solution, he commenced to operate. He said that the advantages which this measure gave him in this case had been incalculable, that as the patient had no pain there was no violent resistance just at the time that perfect quiet was so essential. The drug had acted, too, as a styptic, and there had been but very little hemorrhage. Now, he said, as he had been enabled to carry out in satisfactory detail every step of the operation, he was satisfied that there would be a good result. The most vigorous antisepsis should be employed in rinsing the mouth and flushing the floor of the nasal vault with peroxide of hydrogen. The patient should be fed exclusively per rectum for four days, in order to give the parts that rest so necessary in repair and tissue union.

The second case was a lad of sixteen years who, six weeks before, sustained a fracture of both bones of the right forearm in the upper third. After union there had been discovered a distinct and marked deflection or bowing of the arm, producing a marked deformity. He said that naturally the parents felt aggrieved with the practitioner who had treated him outside, but he had assured them that the fault lay with them probably as much or more than with the doctor, in not carrying out his directions. He had advised operation in this instance for several reasons. The first was, that though the arm would probably preserve fair strength as it was, yet this deformity was a blemish, and later in life would, in physical examination, exclude him from various useful occupations. Further, he advised it because of the comparative simplicity and safety of making a simple or compound fracture in a child, but he would warn the junior members of the profession not to undertake an analogous procedure on the shaft of an adult, unless they wished to wantonly jeopardize the life of their patient or leave him with a stiff, ununited, wasted limb. Nature, he said, was kind to the young, particularly in the repair of osseous structures; but have a care what you do with the healthy medullary cavity of the adult. As union had not solidified here, he said, the chisel easily went through the soft cartilaginous callous, and full correction was a simple matter.

The third patient presented for operation was a child, a girl of four years of age. This little one, six months before, sustained a very serious burn in the palm of the hand. The mother, an honest, hard-working woman, supposing it was a simple matter, treated the case with the ordinary domestic remedies for burns, with the result that all the fingers are firmly locked to the volar surface of the hand by tight, firm, unyielding adhesions. Now, he said, the question arose as to what was to be done? The liberation of the fingers would be a small matter, but the difficulty came in clothing the denuded anterior surfaces and the palm of the hand with integument. One might say at once, employ a Thiersch graft. But a shaving of the scarf-skin will not do here, where so much friction and motion must constantly be borne. I have recommended that Guermonpriz's procedure be employed here in the presence of so much loss of the integument, viz., the sacrifice of one of the fingers, which, after having all the phalanges and tendons dissected out, may be reflected and utilized to fill in the gap; but the father would not consent to this, hence we must do the best we can. In operating each finger was separately, cautiously lifted out of its bed of adhesion and straightened, where a large uncovered breach in the palm was left. To fill this in, a flap with a broad pedicle attached, was carried up from the anterior surface of the wrist and embedded in the gap, its edges being carefully coaptated with fine silk suture. The most thorough asepsis marked every step of the operation. The doctor said the future would largely depend on the preservation of the graft and its assimilation with the tissues in its new situation.

P. S.—Two weeks after operation hiatus in palate was restored, perfect restoration of outline of wrist, and little one's hand gave promise of good result.

### THE VALUE OF ALBUMINURIA AS A MEANS OF DIAGNOSIS.

In a paper on this subject in the *International Monthly Magazine*, Dr. F. R. Sturgis, of New York, concludes from a survey of the literature that albumen in the urine does not necessarily signify any renal disease; that it exists temporarily in many diseases unassociated with any organic renal complication; that from the uncertainty of tests and methods of testing it loses a great deal of its value as a diagnostic sign; and that if present in even a small quantity it is a danger-signal, and if persistent indicates some serious organic lesion.—*Med. and Surg. Reporter.*

